# The transcript interactome of skeletal muscle RNA binding protein motif 3 (RBM3)

**DOI:** 10.14814/phy2.15596

**Published:** 2023-02-07

**Authors:** Zachary R. Hettinger, Amy L. Confides, Peter W. Vanderklish, Esther E. Dupont‐Versteegden

**Affiliations:** ^1^ Department of Physical Therapy, College of Health Sciences University of Kentucky Lexington Kentucky USA; ^2^ Center for Muscle Biology University of Kentucky Lexington Kentucky USA; ^3^ Department of Molecular Medicine, Scripps Research California La Jolla USA; ^4^ Present address: Department of Physical Medicine & Rehabilitation Harvard Medical School Boston Massachusetts USA

**Keywords:** cold shock protein, mRNA half‐life, RIP‐seq, RNA binding proteins

## Abstract

Post‐transcriptional regulation of gene expression represents a critical regulatory step in the production of a functional proteome. Elevated expression of post‐transcriptional regulator RNA binding motif protein 3 (RBM3), an RNA binding protein in the cold‐shock family, is positively correlated with skeletal muscle growth in adult mice. However, mechanisms through which RBM3 exerts its effects are largely unknown. The purpose of this study was to perform RNA immunoprecipitation followed by RNA sequencing (RIP‐seq) and apply a network science approach to understand biological processes (BPs) most associated with RBM3‐bound mRNAs. In addition, through nucleotide‐sequence‐scanning of enriched transcripts, we predicted the motif for skeletal muscle RBM3 binding. Gene set enrichment analysis followed by enrichment mapping of RBM3‐bound transcripts (fold change >3; *p.adj* <0.01) revealed significant enrichment of BPs associated with “Contractile apparatus,” “Translation initiation,” and “Proteosome complex.” Clusters were driven largely by enrichment of *Myh1* (FC: 4.43), *Eif4b* (FC: 5.03), and *Trim63* (FC: 5.84), respectively. Motif scanning of enriched sequences revealed a discrete 14 nucleotide‐wide motif found most prominently at the junction between the protein coding region's termination sequence and the start of the 3′ untranslated region (UTR; *E*‐Value: 1.1 e^−015^). Proof of concept investigation of motif location along enriched transcripts *Myh1* and *Myl4* revealed 3′ UTR binding, suggesting RBM3 involvement in transcript half‐life regulation. Together, these results demonstrate the potential influence of RBM3 in reshaping the skeletal muscle proteome through post‐transcriptional regulation of mRNAs crucial to muscle adaptations.

## Graphical abstract

Skeletal muscle RNA binding protein motif 3 (RBM3) has been recognized as a potential regulator of muscle size, while potential mechanisms are unknown. This study isolates and characterizes the RBM3‐bound transcriptome of resting C2C12 myotubes, and identifies potential transcripts associated with muscle size, which can be leveraged for future and more targeted research.

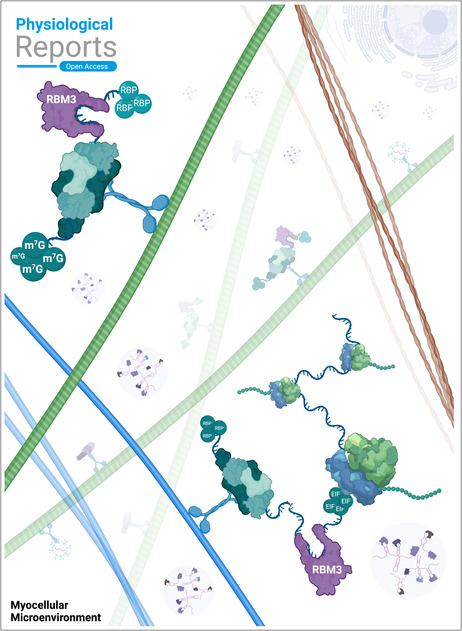

## 1 INTRODUCTION

RNA binding proteins (RBPs) are involved in all RNA processing mechanisms, including alternative splicing, chemical modification, nucleocytoplasmic trafficking, RNA stability, cellular sequestration, and translation initiation and elongation (Gerstberger et al., 2014). As such, approximately 8% of all protein coding genes are directly associated with binding and processing of RNA (Gerstberger et al., 2014), indicating that RBPs play an important role in cellular homeostasis. In the skeletal muscle, the role of RBPs has mainly been investigated during myogenesis, regeneration and neuromuscular diseases and the cumulative evidence indicates that RBPs are critical regulators of these processes (Weskamp et al., 2021). Among the multitude of molecular functions RBPs may utilize to influence these processes, regulation of mRNA stability and their promoted translation has been shown to be important for adult muscle homeostasis (Van Pelt et al., 2019). For example, the stability of transcripts encoding mitochondrial biogenesis genes was shown to be important in the response to chronic contractile activity and the RBPs HuR and AUF1 were directly associated with the decay rates of mitochondrial determining factors (Freyssenet et al., 1999; Lai et al., 2010). However, while these and other data implicate RBPs as central regulators of muscle biology in physiological and pathophysiological contexts (D'Amico et al., 2019; Hausburg et al., 2015; Hosokawa et al., 2019), it is currently unclear how RBPs sculpt the muscle proteome during the processes of hypertrophy and atrophy that dictate muscle mass in these contexts.

RNA binding motif protein 3 (RBM3) is an RBP of the cold shock family (Zhu et al., 2016) and is involved in the maintenance of muscle size by inhibiting atrophy as well as inducing hypertrophy (Van Pelt et al., 2018). These anabolic actions of RBM3 in the muscle most likely relate to its ability to enhance global protein synthesis and alter miRNA levels (Dresios et al., 2005; Pilotte et al., 2011); however, its exact role is still unclear. It is currently unknown which mRNAs are involved in the regulation of muscle size by RBM3 and to which motifs in transcripts RBM3 binds in the skeletal muscle. Indeed, an important first step toward understanding the dynamic roles of RBM3, a thorough characterization of the homeostatic RBM3–mRNA interactome is required. Therefore, the goal of the current study is to identify mRNAs that are associated with RBM3 and determine motifs for binding. Using differentiated C_2_C_12_ myotubes as a model system, we performed RNA immunoprecipitation followed by next generation sequencing (RIP‐seq) to identify transcripts associated with RBM3 and determine if functionally related groups of transcripts are enriched among RBM3 target mRNAs.

## 2 METHODS

### 2.1 Cell culture

The purpose of our study was to identify transcripts bound to RBM3 under resting homeostatic conditions using C_2_C_12_ myotubes as a model system, while information learned here can later be leveraged in primary cell culture for more targeted analyses. Fully differentiated C_2_C_12_ myotubes were grown as described (Ferry et al., 2011). Briefly, C_2_C_12_ myoblasts (ATCC) were grown in growth medium (GM: 10% fetal bovine serum, FBS) (Gibco), 1% penicillin/streptomycin (P/S, Gibco) in Dulbecco's modified eagle medium (DMEM; Gibco) until reaching approximately 85%–90% confluency. At near confluency, GM was replaced with differentiation media (DM: 2% horse serum (Gibco), 1% P/S (Gibco), DMEM) which was replaced every other day for 5 days until myotubes reached full differentiation. A total of three technical replicates per condition were prepared for RIP‐seq.

### 2.2 RIP‐seq protocol

The MagnaRIP RNA‐Binding Protein Immunoprecipitation Kit™ was used to enrich skeletal muscle RBM3‐bound mRNAs (Millipore; cat. no. 17‐700). C_2_C_12_ myotubes were washed with ice‐cold PBS and scraped into microcentrifuge tubes for centrifugation at 1500 rpm for 5 min at 4°C. Lysates were prepared by resuspending cell pellets with RIP lysis buffer (Millipore) and incubated 5 min at 4°C. Two 20 μl aliquots were stored at −80°C for RNA quality assessment and input referencing for sequencing. Magnetic bead‐antibody complexes were prepared by washing beads with RIP wash buffer twice prior to 30 min incubation at room temperature (RT) with 5 μg anti‐RBM3 (Pilotte et al., 2009, 1:500) and normal IgG antibodies (Millipore; cat. no. 17‐700; 1:250). The use of normal IgG antibodies was to ensure that our anti‐RBM3 antibody displayed specificity when binding RNA. Magnetic bead‐antibody complexes were added to a magnetic separator (Millipore) for removal of the supernatant. Complexes were washed and incubated with RIP Immunoprecipitation Buffer (Millipore) and cell lysates overnight rotating at 4°C. Samples were centrifuged and magnetically separated for removal of supernatant and were washed a total of six times prior to purification of RBM3‐bound mRNA. Proteinase K was used to digest bound RBM3 (Millipore). Briefly, a cocktail containing RIP wash buffer, sodium dodecyl sulphate (SDS; Sigma Aldrich), and proteinase K was prepared prior to incubation with immunoprecipitate for 30 min rocking at 55°C. Following protein digestion, samples were washed three times and combined with phenol:chloroform:isoamyl alcohol (Millipore) and centrifuged at 14,000 rpm for 10 min at RT. The aqueous layer containing RNA was isolated and combined with Salt Solutions I and II, Precipitate Enhancer (Millipore), and 100% ethanol (EtOH; Sigma) for precipitation at −80°C overnight. Samples were centrifuged at 14,000 rpm for 30 min at 4°C. RNA pellet was washed with 80% EtOH and centrifuged at 14,000 rpm for 15 min at 4°C. Samples were air‐dried and resuspended in RNase‐free water for quality control (QC) analyses and sequencing. An Agilent Bioanalyzer was used to verify RNA integrity numbers (RIN) of 8 or higher (Agilent) prior to cDNA library generation and sequencing on an Illumina Novaseq 6000 platform, yielding a minimum of read depth of 20 million reads/sample, and read length of 150 base pairs (Novogene).

### 2.3 Gene set enrichment mapping

Following QC of raw reads, trimming, and alignment (Langmead & Salzberg, 2012), as well as peak characterization using HOMER, RBM3‐bound targets were filtered (*FC* >3.0; *FDR adjusted p* < 0.01) and analyzed using Gene Set Enrichment Analyses (GSEA; Raudvere et al., 2019) and Enrichment Map (Merico et al., 2010; Reimand et al., 2019) using Cytoscape (RRID: SCR_003032). Briefly, RBM3‐bound targets were entered as an ordered query and the data sources “Biological Processes” and “Reactome” were selected as data outputs. Data were corrected for multiple comparisons (FDR adjusted *p* < 0.01) as well as term size (5–500) to reduce term redundancy (Reimand et al., 2019). The resulting .gem file was then imported into Cytoscape for mapping of enriched biological processes (Reimand et al., 2019). Following filtration for significantly enriched processes (node cutoff: FDR adjusted *p* < 0.05; edge cutoff: Jaccard Score (similarity) < 0.05), an enrichment map representing the biological processes of RBM3‐bound targets was generated for visualization and interpretation.

### 2.4 RNA binding motif prediction

Multiple Em for motif elicitation (MEME) was used to predict and characterize the preferential RNA binding motif(s) of skeletal muscle RBM3 (MEME Suite—Motif‐based sequence analysis tools RRID:SCR_001783). Briefly, MEME analyzes recurring and ungapped sequence patterns within sequencing data and reports the best‐fit width, number of occurrences, and description of the predicted motif (Bailey & Elkan, 1994). As such, RBM3‐enriched reads (FC >3.0; FDR adjusted *p* < 0.05) were imported into the MEME Suite (https://meme‐suite.org/meme/), and an *E*‐value threshold of 0.05 was used to filter for significantly enriched motifs (Tanaka et al., 2014).

### 2.5 Statistics

Fold changes were calculated by determining the abundance of RBM3‐bound mRNAs relative to the input mRNA from control C_2_C_12_ myotubes. Statistical significance for enriched genes was set at *FDR p* adjust <0.01 and FC >3.0 to account for the possibility of non‐specific interactions between RBM3 and other binding proteins. Predicted motifs using MEME were considered significant at *E* < 0.05.

### 2.6 Rigor and reproducibility

Each of the described experiments meet the Rigor and Reproducibility guidelines provided by the American Physiological Society. Three biological replicates were used for both control and IP cell culture conditions. The use of IgG control antibodies (Millipore; cat. no. 17‐700; 1:250) was used to ensure specificity of anti‐RBM3 antibody. Purified RNA with RIN scores of 8 or higher were used for sequencing. A total of three technical replicates were provided for sequencing. Sequencing and library preparation was performed by an unbiased and blinded third party (Novogene). To analyze only the most enriched genes, a FC of greater than 3.0 and a *q*‐value of less than 0.01 was used for enrichment analyses.

## 3 RESULTS AND DISCUSSION

### 3.1 Enrichment mapping of RBM3‐bound mRNA suggests roles in skeletal muscle adaptations

**FIGURE 1:**
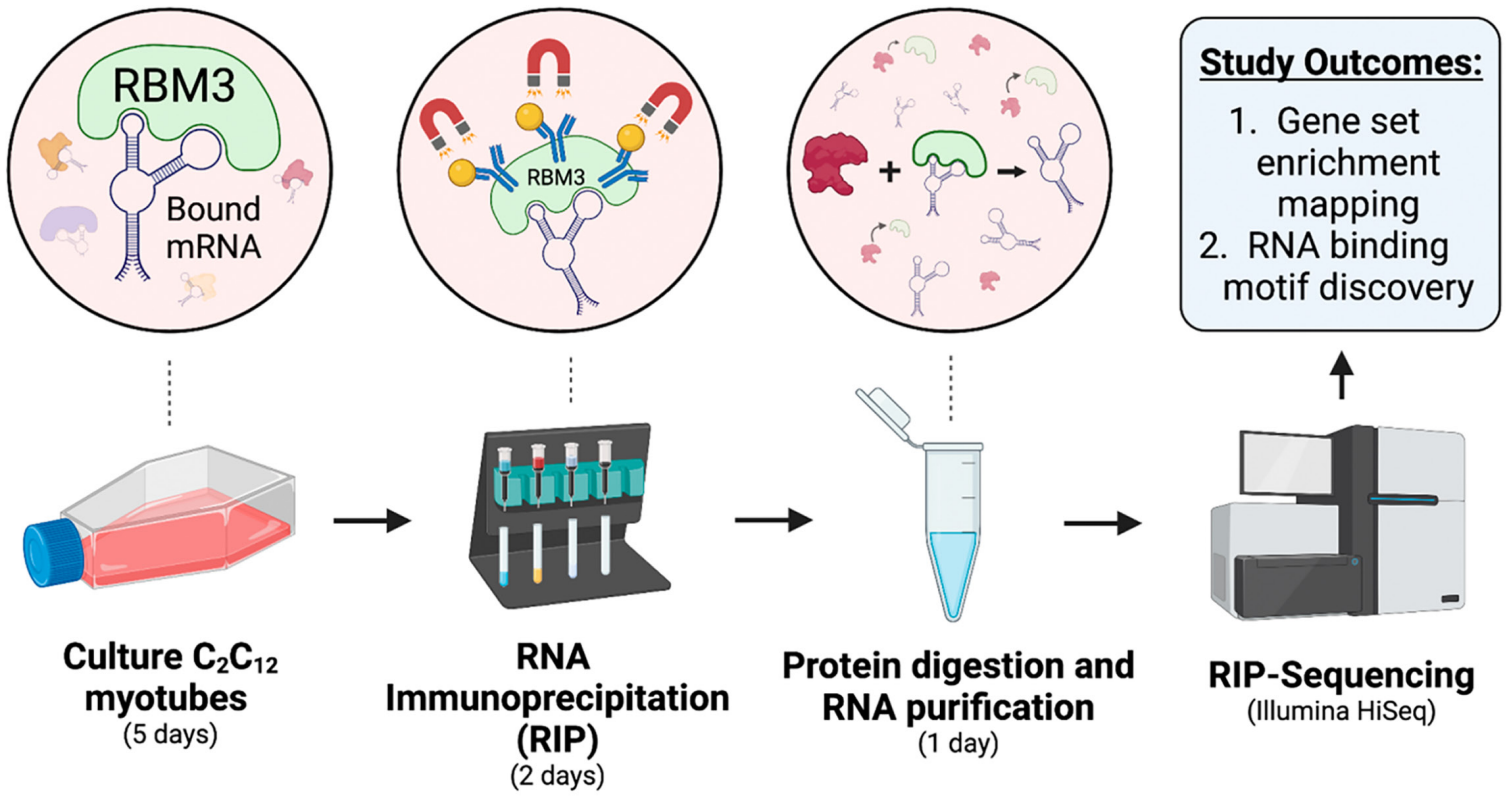
Experimental design. C_2_C_12_ myotubes at 5 days of differentiation were used for RIP‐seq experiments using previously validated anti‐RBM3 antibody (Pilotte et al., 2009). *N* = 3 biological replicates per condition (IP or IgG control).

We have previously shown the expression levels of RBM3 closely track with changes in muscle size (Dupont‐Versteegden et al., 2008), both during hypertrophy and following disuse atrophy (Van Pelt et al., 2018), leading us to question what transcripts are bound to RBM3, and how does this associate with changes in muscle size. Therefore, as a first step toward characterizing the RBM3 interactome in the skeletal muscle, we used C_2_C_12_ myotubes as a model system to characterize which transcripts RBM3 binds under normal resting conditions (Figure 1). Indeed, we have previously shown RBM3 overexpression and knockdown in C_2_C_12_ myotubes to recapitulate in vivo situations of muscle hypertrophy and atrophy, respectively, which demonstrates the utility of C_2_C_12_ myotubes as an acceptable model system for the current investigation (Van Pelt et al., 2018). Immunoprecipitation of the muscle RBM3‐bound transcriptome yielded a total of 2933 unique transcripts (out of approximately ~12 k transcripts). We performed GSEA using gProfiler (https://biit.cs.ut.ee/gprofiler/gost) and enrichment mapping using Cytoscape (https://cytoscape.org/) to reveal mechanistic insights into the role of RBM3 in the skeletal muscle. GSEA revealed the most common molecular functions of RBM3‐bound transcripts to be associated with *Translation Factor Activity*, *Heat Shock Protein Binding*, and *RNA Cap Binding*. The most common cellular compartment of RBM3‐bound transcripts was found to be at *Nuclear Envelope*, *Contractile Fiber*, the *Extracellular Exosome*. Lastly, the most enriched biological processes of the gene set included *Muscle Cell Development*, *Protein Stabilization*, and *Regulation of Intracellular Transport*.

**FIGURE 2:**
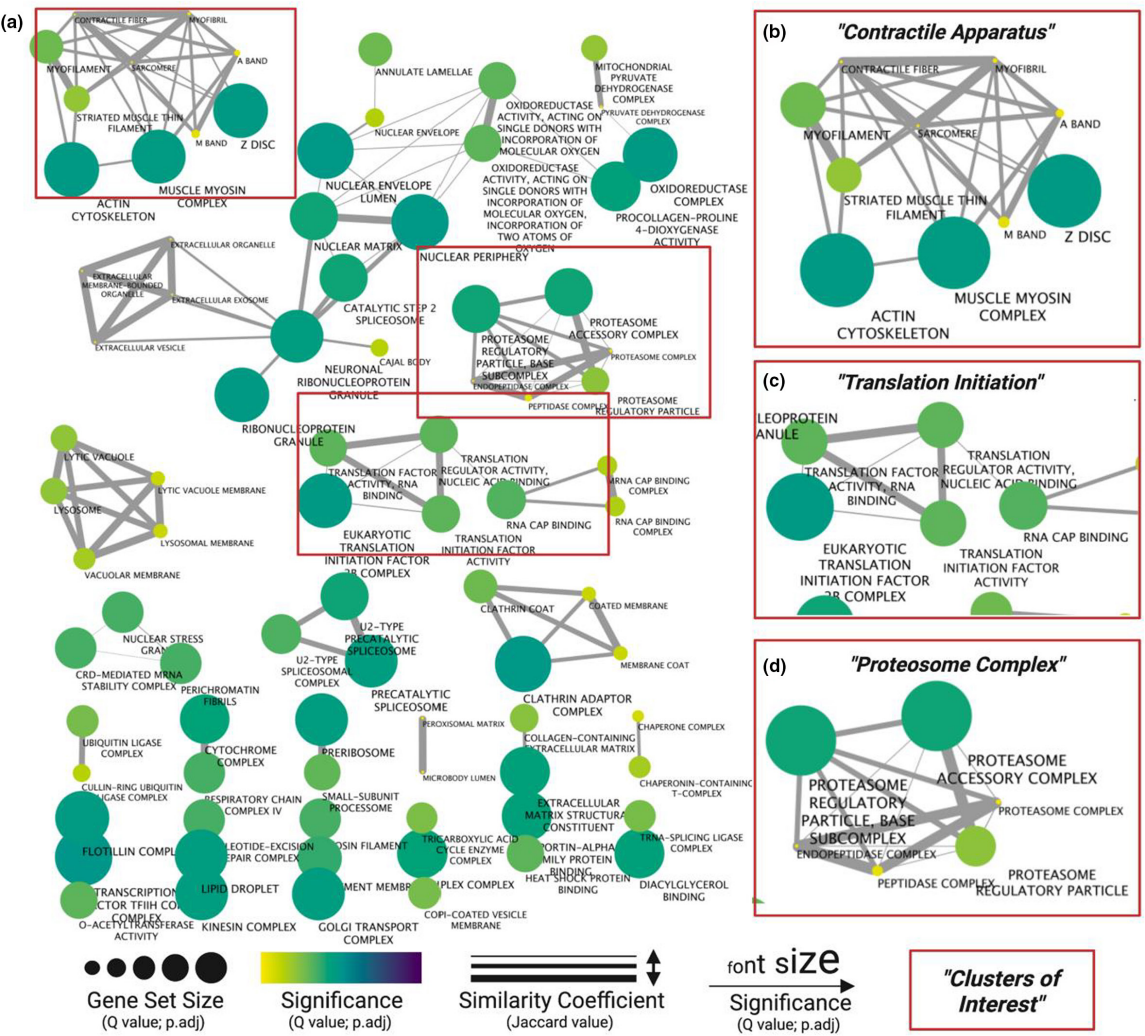
Enrichment map of biological processes related to skeletal muscle RBM3‐bound mRNAs. (a) Enriched genes (FC >3; *q*‐value <0.01) were used for GSEA using gProfiler, and then clustered and visualized using Cytoscape. Biological processes with overlapping gene sets are connected by edges. Node color and label font size indicate significance of enriched gene set. Edge width corresponds to the similarity coefficient between gene sets. Node size corresponds to gene set size. Significantly enriched ‘hubs’ of (b) *Contractile Apparatus*, (c) *Translation Initiation*, and (d) *Proteosome Complex* are highlighted.

GSEA analyses provide a useful approach to identifying common threads in gene set enrichment data but can both over and underestimate enriched processes due to variation in gene set size. To overcome this obstacle, we used Enrichment Map of GSEA results to reveal and simplify the most consistently enriched processes of RBM3‐bound RNAs. Using a similarity coefficient to account for gene set overlap, along with statistical significance of each process, revealed a hierarchal summary of processes with “Contractile Apparatus” (Figure 2a,b), “Translation Initiation” (Figure 2a,c), and “Proteosome Complex” (Figure 2a,d) presenting the most highly enriched clusters. The individual elements of the top cluster included terms such as *Muscle Myosin Complex* and *Z Disc*, mediated in part by enrichment of genes including myosin heavy chain 1 (*Myh1*), alpha‐actinin‐2 (*Actn2*), and integrin subunit beta 1 binding protein 2 (*Itgb1bp2*) (Figure 2a,b; Table 1). The term “Proteosome complex” displayed significant overlap of gene sets including *Proteosome regulatory particle* and *Proteosome complex*, mediated in part by enrichment of protein degradation marker genes such as ubiquitin 4 (*Ubqln4*) and 26 S proteasome non‐ATPase regulatory subunit 7 (*Psmd7*) (Figure 2c; Table 1). *Ribonucleoprotein Granule*, mediated by enrichment of heterogeneous nuclear ribonucleoproteins A2/B1 (*Hnrnpa2b1*), served as a bridge between numerous clusters associated with RNA metabolism and transport, including *Cajal Body* (Ubiquitin Specific Peptidase Like 1; *Uspl1*), *Spliceosome* (splicing factor 3b subunit 1; Sf3bp1), and *Extracellular Exosome* (*Anxa1*) (Figure 2a; Table 1). Lastly, there was significant enrichment of numerous eukaryotic initiation factors (EIFs), including *Eif2b*, *Eif3f*, and *Eif4b*, constituting a “Translation Initiation” cluster constituted by terms such as *Translation Factor Activity—RNA Binding*, *Eukaryotic Translation Initiation 2B Complex*, and *RNA Cap Binding* (Figure 2a,d; Table 1).

**TABLE 1:** Gene set enrichment summary of biological processes most associated with RBM3‐bound mRNA.

Summary description	Gene set description(s)	Gene	*E‐*score
“Proteosome Complex”	“Proteosome accessory complex”; “Proteosome complex”; “Proteosome regulatory particle”; “Peptidase complex”; “Endopeptidase complex”; “Proteosome regulatory particle, base subcomplex”	*Psmd11*	3.35
		*Psmd3*	3.17
		*Rad23b*	**6.56**
		*Ubqln4*	**5.54**
		*Adrm1*	3.00
		*Psma3*	**5.93**
		*Psmd7*	**7.50**
		*Psma6*	4.82
		*Ubr4*	4.76
“Translation Initiation”	“Translation Regulator Activity, Nucleic Acid Binding”; “Translation Initiation Factor Activity”; “Translation Factor Activity, RNA Binding”	*Eif3l*	3.29
		*Eif4e2*	**7.55**
		*Eif4b*	**5.03**
		*Eif3d*	4.76
		*Gfm1*	4.76
		*Eif2b1*	4.63
		*Eif1ax*	4.36
		*Gstp1*	3.45
		*Eif2s2*	4.19
		*Eif3f*	4.14
		*Larp1*	4.10
	“Eukaryotic Translation Initiation Factor 2B Complex”	*Eif2b1*	4.63
		*Eif2b5*	3.36
		*Eif2b2*	4.07
“Contractile Apparatus”	“Actin Cytoskeleton”; “Myofilament”; “Striated Muscle Thin Filament”; “Z Disc”; “Muscle Myosin Complex”; “M Band”; “Sarcomere”; “Contractile Fiber”; “Myofibril”; “A Band”	*Flnc*	4.76
		*Myl1*	4.43
		*Vps11*	3.52
		*S100a1*	4.36
		*Myl6*	4.73
		*Actn4*	**7.01**
		*Itgb1bp2*	**6.71**
		*Actn2*	**5.98**
		*Myom1*	**5.93**
		*Myom2*	**5.43**
		*Trip6*	**5.53**
		*Myl4*	**7.86**
		*Dnaja3*	4.85
		*Ttn*	**5.26**
		*Trim63*	**5.84**
		*Tnnt2*	**7.81**
		*Tpm1*	4.60
		*Dhx9*	**5.14**
		*Fbxl22*	4.36

*Note*: Gene set descriptions represent individual gene ontology terms. *E‐*score represents enrichment of genes in the immunoprecipitated group relative to the input control. Genes highlighted in bold indicate greater than fivefold enrichment (*q* < 0.01). *N* = 3 technical replicates per condition (IP or IgG control).

RBM3 is known to promote proliferation (Ferry et al., 2011), enhance global protein synthesis (Dresios et al., 2005), and prevent cell death following hypoxic and/or cytotoxic conditions (Chappell et al., 2001; Ferry et al., 2011; Van Pelt et al., 2018). We reported higher expression levels of RBM3 in the skeletal muscle during disuse atrophy, particularly in association with the loss of cross sectional area, suggesting a compensatory role for RBM3 to counteract muscle loss (Dupont‐Versteegden et al., 2008). Indeed, when overexpressed RBM3 induces muscle hypertrophy and attenuated atrophy (Van Pelt et al., 2018). Our data in the current study suggest mechanisms through which RBM3 modulates muscle size regulation. A crucial determinant in muscle homeostasis is the balance between protein degradation and synthesis (Bodine, 2013; Miller et al., 2019) and our data indicate that RBM3 may influence both processes as both sarcomeric molecules including *Ttn* and *Myh1*, and protein degradation molecules including *Fbxl22* and *Trim63* (Murf1), were each significantly enriched with RBM3 IP. In addition, evidence of an anabolic influence in protein synthesis was found in the enrichment of numerous translation initiation factors including *Eif4b* and *Eif2b1*, among numerous other subunits of eIF2 and eIF3. The enrichment of eIF subunits as well as previous reports implicating compensatory role of RBM3 in muscle mass regulation are altogether suggestive of the integrated stress response (ISR). The ISR is an evolutionarily conserved mechanism that cells use in situations of stress, through post‐transcriptionally and post‐translationally remodeling the cell to return to homeostasis (Pakos‐Zebrucka et al., 2016). It is possible that RBM3 assists in coordinating the ISR to restore muscle mass following atrophy‐inducing stimuli. However, this requires further investigation. Lastly, RBM3 was enriched with transcripts related to ribonucleoprotein granule colocalization, including Ddx1 and Hnrnpa2b1; thus, RBM3 may regulate the abundance of, and be found complexed with machinery necessary for protein synthesis (Dresios et al., 2005). Indeed, it has been suggested that a large percentage of mRNA is degraded by decay pathways in the nucleus (~40%), and that a critical determinant to ensuring nuclear to cytoplasmic transport involves specific mRNA sequences for RBP binding (Houseley & Tollervey, 2009; Smalec et al., 2022). Therefore, RBPs such as RBM3 may ensure delivery of mRNAs to cytosolic ribosomes for translation, and context‐dependent degradation of transcripts in situations of cellular stress. Together, our data highlight the diverse range of potential processes associated with RBM3‐mRNA binding in the skeletal muscle.

### 3.2 Binding motif of RBM3 reveals potential regulatory roles in transcript stability

**FIGURE 3:**
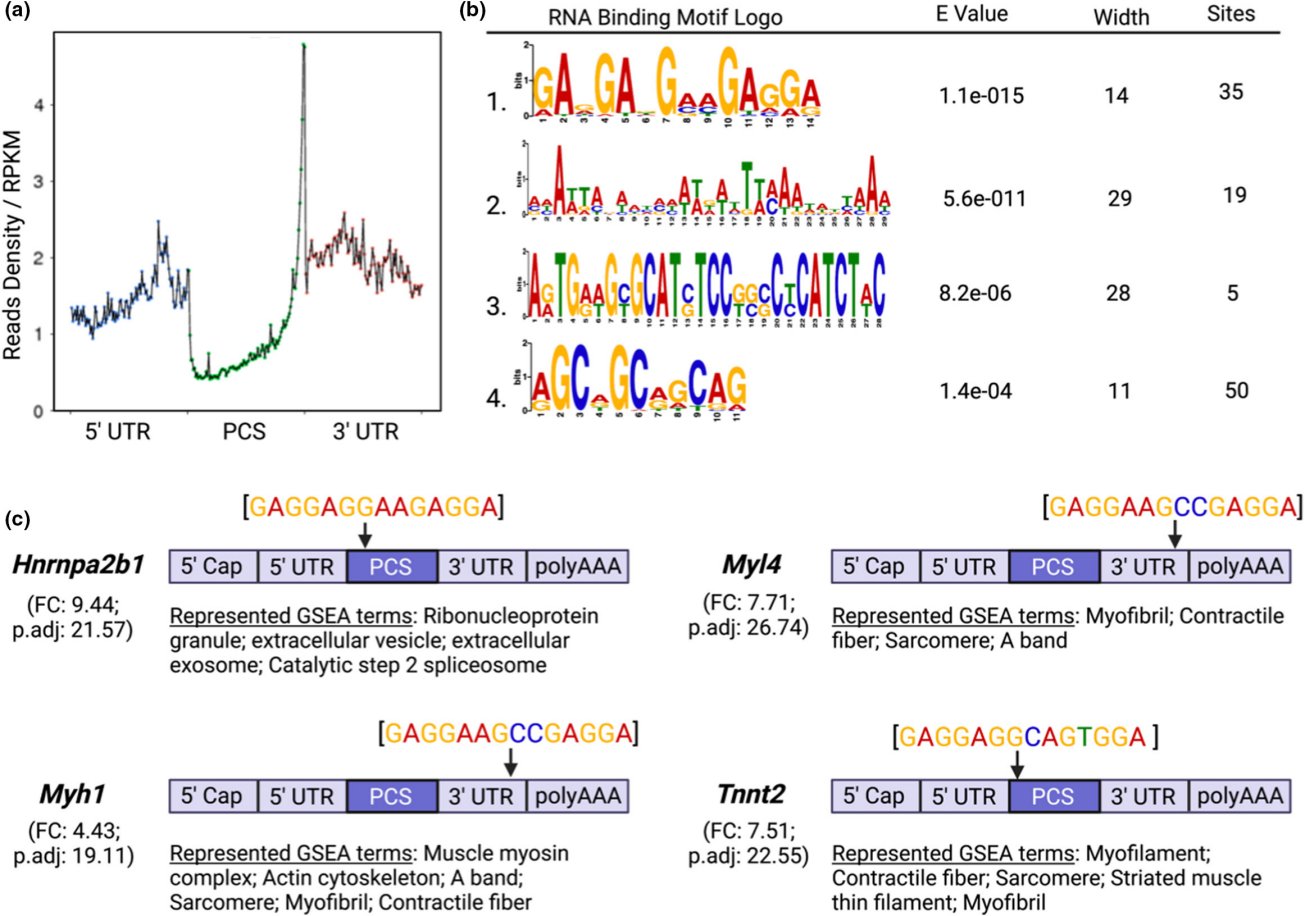
RNA binding motif analysis of skeletal muscle RBM3. (a) Read density of RBM3 binding relative to the reads per kilobase of transcript. (b) RNA binding motifs of top predictions using the motif‐based sequence analysis suite MEME of RBM3‐enriched transcripts. *E < 0.05. (c) Identification of motif sequence location on RBM3‐enriched transcripts using top predicted binding motif sequences.

To better understand the potential regulatory role of RBM3, we sought to determine the nucleotide sequence motif common to transcripts enriched in RIP‐seq data. Characterization of binding along the gene sequence of RBM3‐enriched transcripts revealed a significant higher degree of binding at the junction between the termination sequence of the protein coding sequence (PCS) and the 3′UTR (Figure 3a). While 3′UTR binding was the most enriched location along the gene, there were significant occurrences of binding in the 5′UTR and, albeit significantly less, the PCS (Figure 3a). The most commonly occurring sequence motif for all RBM3‐enriched mRNAs included a discrete GA‐rich 14‐nucleotide wide motif, followed by longer and far less significant motifs (Figure 3b). As proof of concept, we selected candidate transcripts from our dataset and investigated where the most statistically significant predicted binding motif lies along the transcript's body. Interrogation of *Hnrnpa2b1*, *Myl4*, *Myh1*, and *Tnnt2* revealed positive hits for the predicted motif at various sites along genes. Both *Myl4* and *Myh1* revealed binding at the 3′UTR near the polyA tail, while the binding motif was found in the PCS of both *Hnrnpa2b1* and *Tnnt2* nearest the start codon for transcript translation (Figure 3c). The GA‐rich region is similar, but not identical, to a region identified by Liu et al. using photoactivatable nucleoside enhanced cross‐linking immunoprecipitation (PAR‐CLIP) that was also most often found in PCS or 3′UTR of immortalized mouse embryonic fibroblasts and 293T cells (Liu et al., 2013). Target genes for this motif were involved in regulating transcription and mRNA metabolic processes and it was shown that RBM3 stabilized its target pre‐mRNAs and RNAs. It is tempting to speculate that RBM3 has a similar RNA stabilizing role for those transcripts to which it binds in the skeletal muscle. However, future studies validating that the specific transcripts identified in this study associate with RBM3, as well as the use of reporter constructs to determine whether the sequence motifs are necessary and sufficient for binding, are needed.

There are a few important considerations for interpretation of the motif data. First, the degree of specificity of RBM3 binding, or lack thereof, can have critical consequences for developing a proteome appropriate the function of muscle. For example, specific binding of RBM3 to sarcomeric mRNAs may ensure cytoplasmic delivery to ribosomes for translation, thereby enhancing muscle size. However, lack of specificity may lead to promiscuous binding of a large proportion of the transcriptome, which has been shown in developmental contexts to be influential in establishing cellular polarity during division (Saha et al., 2016). The enrichment of mRNAs involved in contractile apparatus, translation initiation, and proteasome complex, indicates that specificity of RBM3 binding to its motifs is likely high in differentiated skeletal muscle. Second, mRNA does not exist in a linear conformation, but rather secondary and sometimes tertiary structures (Mauger et al., 2019). Stem‐loop structures are typically found in the 3′UTR of mRNA, caused by a specific combination of bonds formed from defined nucleotide sequences (Penno et al., 2015). It has been postulated that RBP binding is not sequence specific, but rather shape/structure dependent (Tan et al., 2013), which is determined by specific nucleotide sequences. Therefore, it is possible that the sequences identified here represent the various mRNA conformations RBM3 prefers, even though the structure of RBM3‐bound mRNA is currently unknown. Understanding the potential contribution of mRNA structure to RBM3 binding will be an important step in elucidating the post‐transcriptional influence of RBM3. An important note for consideration is that while human and mouse genomes have been shown to be nearly indistinguishable, this does not hold true for human and mouse transcriptomes. Indeed, a vast collection of studies have demonstrated species‐specific differences in alternative splicing, which has been postulated to contribute to the evolution of species through diversification of the proteome (Barbosa‐Morais et al., 2012). Therefore, it is likely that the RBM3 nucleotide binding motif identified here is different in humans. However, while the nucleotide sequence preference of RBM3 may differ between mouse and human transcripts, the location of binding along the transcript, and therefore the regulatory function, may be conserved. Future studies in human cells will help to answer these questions. Lastly, RBP binding to specific nucleotide sequences is influential in production of mature mRNA at the spliceosome (Cho et al., 2011; Stagsted et al., 2021). Indeed, it has been shown that alterations in the expression of various RBPs leads to tissue‐specific splicing patterns (Kashima et al., 2007; Venables et al., 2008), and low binding specificity can lead to targeted degradation of mRNAs. In our data, we found that many RBM3‐enriched genes are characterized by subcellular location at the nuclear periphery (Figure 2a), sites commonly associated with spliceosome colocalization. Moreover, enrichment mapping of transcript‐associated biological processes revealed numerous clusters related to the spliceosome, such as the *Catalytic Step 2 Spliceosome*, and the *Precatalytic Spliceosome*. These results suggest a role for RBM3 in both the production of mature mRNA and the shuttling of mRNA from the nucleus to the cytoplasm, explaining the presence of RBM3 in both compartments (Smart et al., 2007).

## 4 CONCLUSIONS AND FUTURE DIRECTIONS

It is becoming increasingly evident that RBPs exert critical post‐transcriptional regulatory influences in the generation of a functional proteome. Our data indicate that RBM3, a stress responsive RBP with demonstrated anabolic influences in the muscle, binds to sets of transcripts functionally enriched in processes crucial to skeletal muscle size regulation, including sarcomere content, proteosome complex, and translation initiation. We propose that RBM3 may represent a key node in adaptive posttranscriptional regulatory processes linked to the maintenance of muscle mass and function.

## AUTHOR CONTRIBUTIONS

Author contributions were determined using the CRediT model: Conceptualization: ZRH, PWV, EED. Methodology: ZRH, ALC. Investigation: ZRH, ALC, PWV, EED. Visualization: ZRH, EED. Funding acquisition: ZRH, EED. Project administration: PWV, EED. Supervision: PWV, EED. Writing—original draft: ZRH, EED. Writing—review & editing: ZRH, ALC, PWV, EED.

## FUNDING INFORMATION

Funding for this study was supported by pilot funding provided by the University of Kentucky's College of Health Sciences Endowed Professor Funds (EDV).

## CONFLICT OF INTEREST STATEMENT

The authors have no conflicts of interest to declare.

## ETHICS APPROVAL

Not applicable.
