# Correction to The transcript interactome of skeletal muscle RNA binding protein motif 3 (RBM3)

**DOI:** 10.14814/phy2.15839

**Published:** 2023-10-27

Hettinger ZR, Confides AL, Vanderklish PW, Dupont‐Versteegden EE. The transcript interactome of skeletal muscle RNA binding protein motif 3 (RBM3). *Physiol Rep*. 2023;11(3):e15596. doi:10.14814/phy2.15596

The formatted Excel file containing the differentially enriched genes used to generate the manuscript's findings was not made readily accessible for readers to download and utilize for their own research. The Excel file has been included here.

We apologize for this error.

## Supporting information


Data S1.

